# MACC1 promotes carcinogenesis of colorectal cancer via β-catenin signaling pathway

**DOI:** 10.18632/oncotarget.1993

**Published:** 2014-05-20

**Authors:** Tiantian Zhen, Sujuan Dai, Hui Li, Yang Yang, Lili Kang, Huijuan Shi, Fenfen Zhang, Dongjie Yang, Shirong Cai, Yulong He, Yingjie Liang, Anjia Han

**Affiliations:** ^1^ Department of Pathology, the First Affiliated Hospital, Sun Yat-Sen University, Guangzhou, China; ^2^ Department of Gastrointestinal Surgery, the First Affiliated Hospital, Sun Yat-Sen University, Guangzhou, China

**Keywords:** MACC1, β-catenin, colorectal cancer, carcinogenesis

## Abstract

Here we confirmed that metastasis-associated in colon cancer 1 (MACC1) and β-catenin expression were higher in colorectal cancer (CRC) cells and tissues than those in normal colonic epithelial cell line and adjacent non-tumour colorectal mucosa (ANM) tissues, respectively. MACC1 expression was significantly related to histological differentiation (p<0.001), UICC stage (p=0.029), T classification (p=0.017), and N classification (p=0.023). Cox regression analysis demonstrated that high MACC1/abnormal β-catenin expression was the strongest independent prognostic indicator for reduced overall survival in CRC patients. Significant positive correlation between MACC1 expression and abnormal β-catenin expression was found in CRC tissues. MACC1 knockdown dramatically inhibited cellular proliferation, migration, invasion, colony formation, and tumorigenesis, both in vitro and in vivo, but induced apoptosis in CRC cells. Further MACC1 over-expression increased Met, β-catenin, and its downstream genes including c-Myc, cyclin D1, and MMP9 expression, and its upstream gene phos-GSK3β (Ser9) expression. In addition, MACC1 increased vimentin and suppressed E-cadherin in HCT116 cells. Silencing of MACC1 reversed all these changes. Our results firstly suggest that MACC1 plays an important role in carcinogenesis and progression of CRC through β-catenin signaling pathway and mesenchymal-epithelial transition.

## INTRODUCTION

Colorectal cancer (CRC) is one of the most common malignancies worldwide. Metastasis-associated in colon cancer-1(MACC1), a newly identified key regulator of hepatocyte growth factor (HGF)-MET signaling, predicts colon cancer metastasis [1-3]. Recently, MACC1 expression has been found in lung cancer [4], hepatocellular carcinoma [5], ovarian carcinoma [6], gastric carcinoma [7], esophageal cancer [8], and nasopharygneal carcinoma [9]. Overexpression of MACC1 associates with the progression of these carcinomas and prognosis of the patients with these carcinomas [4, 7, 8].

The Wnt/β-catenin signaling pathway could be regulated by other signaling molecules or pathways, including a destruction complex consisting of casein kinase Iα (CKIα), glycogen synthase kinase 3β (GSK3β), adenomatous polyposis coli (APC) and Axin. Phosphorylation of β-catenin by GSK-3β results in ubiquitin-mediated degradation of β-catenin, reducing translocation of β-catenin into the nucleus. Consequently, the transcription of many proto-oncogenes, such as cyclin D1, c-Myc, and human telomerase reverse transcriptase is dramatically suppressed [3, 10-14]. Recently,

Our recent study has reported that MACC1 down-regulation inhibits proliferation and tumourigenicity of nasopharyngeal carcinoma cells through Akt/beta-catenin signaling pathway [9]. To our knowledge, there is no report on MACC1 regulating β-catenin signaling pathway in CRC. Our current study is to investigate whether MACC1 regulates β-catenin signaling pathway in CRC and the underling mechanism.

## RESULTS

### MACC1 and β-catenin expression in CRC cell lines and fresh CRC tissues

**Figure 1:**
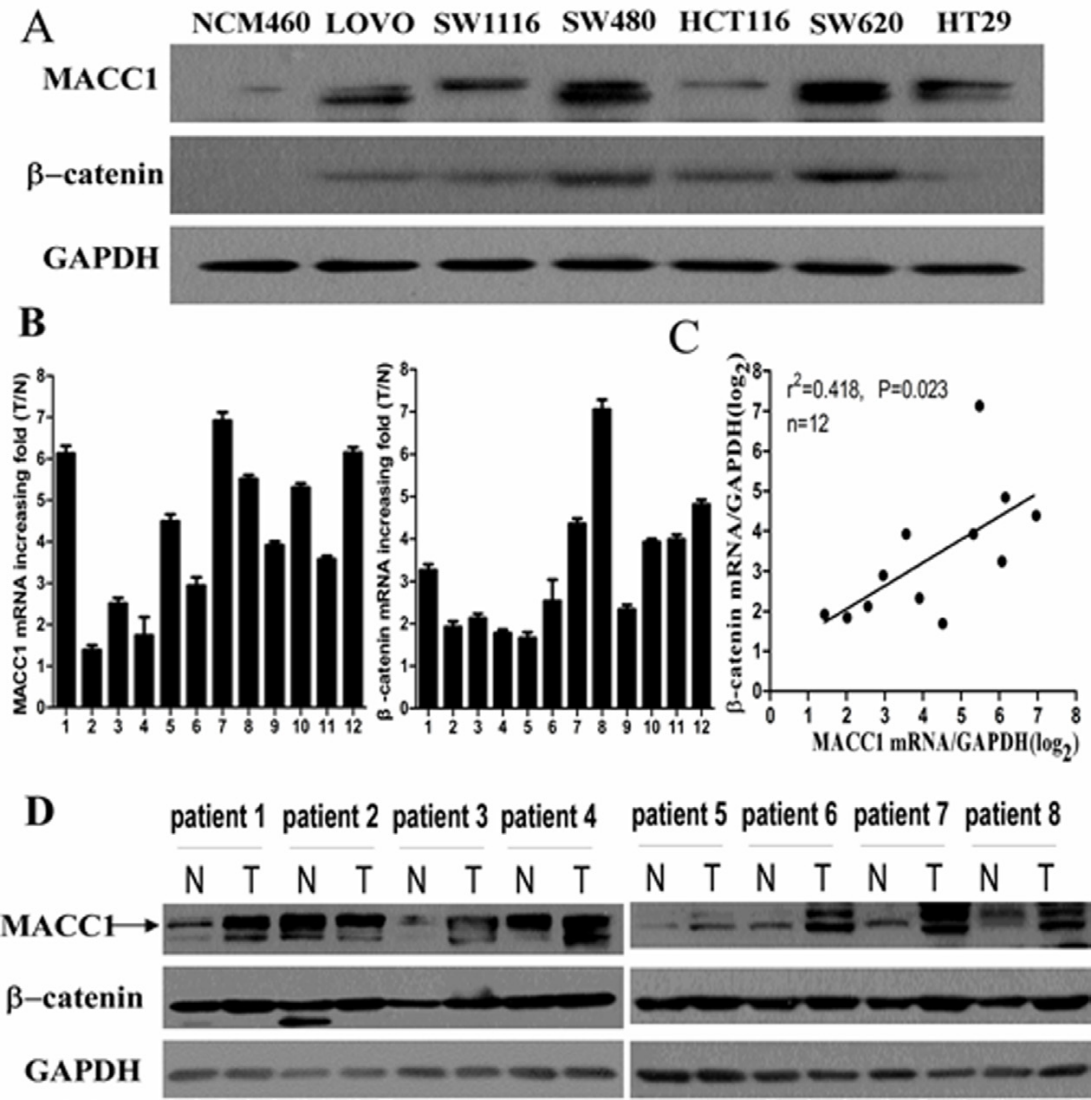
MACC1 protein expression in CRC cell lines (LOVO, SW1116, SW480, HCT116, SW620, and HT29) and normal colonic mucosa epithelial cell (NCM460) by western blot analysis (A); MACC1 and β-catenin mRNA expression in 12 pairs of fresh CRC and adjacent non-tumour colorectal mucosa (ANM) tissues by real-time PCR analysis (B); Significant positive correlation between MACC1 and β-catenin mRNA expression in such 12 pairs of fresh CRC and ANM tissues (C); MACC1 and β-catenin protein expression in 8 pairs of fresh CRC and ANM tissues by western blot analysis (D).

MACC1 and β-catenin protein expressions were higher in CRC cell lines including LOVO, SW1116, SW480, HCT116, SW620, and HT29 compared with human colonic epithelial cell line NCM460 by western blot analysis (Figure 1A). Real-time PCR showed that MACC1 and β-catenin mRNA expression were significantly higher in 12 samples of fresh CRC tissues compared to their respective ANM tissues. The scatter diagram showed that there was a significantly positive correlation between MACC1 and β-catenin mRNA expression in such 12 samples of fresh CRC tissues (p =0.023, Figure 1B-C). Likewise, MACC1 and β-catenin expression were dramatically increased in eight samples of fresh CRC tissues compared with their respective ANM tissues by Western blot analysis (Figure 1D).

### MACC1 and β-catenin expression in paraffin-embedded CRC tissues and its relationship with clinicopathological features of CRC

**Figure 2:**
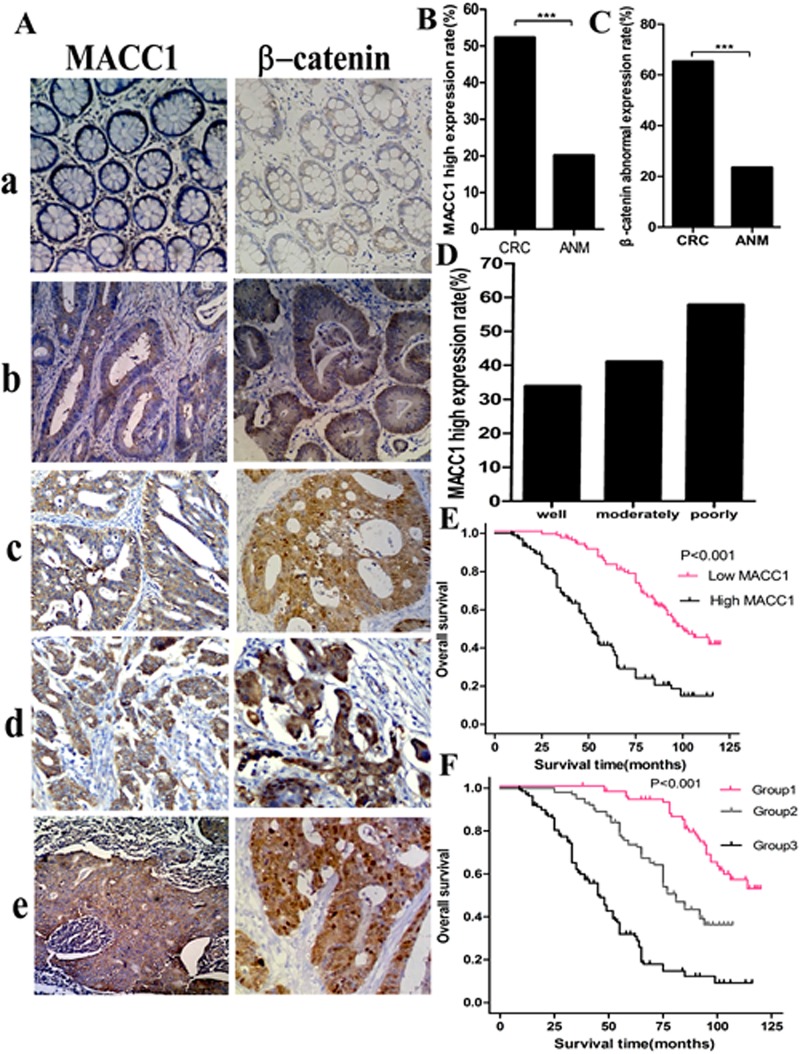
MACC1 and β-catenin expression in ANM (a) and CRC (b, well differentiated CRC; b, moderately differentiated CRC; d, poorly differentiated CRC; e, lymph node metastatic CRC) tissues by immunohistochemistry staining ×200 (A); MACC1(B) and abnormal β-catenin (C) expression was significantly higher in CRC than that in ANM, respectively ***p<0.001; high MACC1 expression increased with the decreased histological differentiation degree of CRC (D); Overall survival(OS)of CRC patients with different levels of MACC1 expression by Kaplan-Meier analysis (E); OS of CRC patients according to the combination of MACC1 and β-catenin expression levels Log-Rank test: Group 1 VS Group 2, p<0.001; Group 1 VS Group 3, p<0.001; Group 2 VS Group 3, p<0.001(F).

In our series, MACC1 positive signals were mostly located in the cytoplasm of CRC cells with minor nuclear distribution by immunohistochemistry staining. Of 323 samples of paraffin-embedded CRC tissues, 169 samples (52.3%) were MACC1 high expression, 154 samples (47.7%) were MACC1 low expression. However, only 65 samples (20.1%, 65/323) were MACC1 high expression in ANM tissues. MACC1 expression was significantly higher in CRC tissues than that in ANM tissues (p<0.001). In addition, MACC1 expression was significantly related to histological differentiation (p<0.001), UICC stage (p=0.029), T classification (p=0.017), and N classification (p=0.023, Figure 2). However, no significant relationship between MACC1 expression and gender, age, and M classification was found (Table 1).

**Table 1:** The relationship between MACC1 expression and the clinicopathological features of CRC

Characteristics		MACC1 expression			Overall survival analysis			
		Low (%)	High (%)	P value	3-year overall survival %	5-year overall survival %	Univerate P value	Multivariate P value
Gender	Male	87 (26.9)	108 (33.4)	0.174	80.6	62.7	0.865	
	Female	67 (20.7)	61 (18.9)		82.1	60.0		
Age	>60	91 (28.2)	88 (27.2)	0.205	81.1	62.9	0.703	
	≤60	63 (19.5)	81 (25.1)		82.4	60.6		
Histological differentiation	Well	43 (13.3)	22 (6.8)	0.006	96.9	82.7	<0.001	0.016
	Moderately	73(22.6)	95(29.4)		84.9	68.3		
	Poorly	38 (11.8)	52 (16.1)		62.5	31.5		
UICC stage	I- II	76 (23.5)	63 (19.5)	0.029	92.7	79.9	<0.001	0.033
	III -IV	78 (24.1)	106 (32.8)		73.5	48.5		
T classification	T1 -T2	52 (16.1)	37(11.5)	0.017	94.3	78.5	0.001	0.004
	T3 -T4	102 (31.6)	132 (40.9)		85.4	48.5		
N classification	N0	86 (26.7)	73 (22.6)	0.023	88.1	68.6	0.015	0.040
	N1 -N2	68 (21.1)	96 (29.7)		75.6	54.7		
M classification	M0	104 (32.2)	127 (39.3)	0.130	86.5	68.1	0.005	0.734
	M1	50 (15.5)	42 (13.0)		69.8	41.9		
MACC1 expression	Low	154(47.7)			96.7	82.8	<0.001	<0.001
	High		169(52.3)		67.8	41.5		
MACC1/β-catenin expression	Group1				100.0	93.8	<0.001	*
	Group2				95.1	73.6		
	Group3				60.6	32.0		

Group 1, Low MACC1/normal β-catenin expression; Group 2, Low MACC1/abnormal β-catenin + high MACC1/normal β-catenin expression; Group 3, High MACC1/abnormal β-catenin expression. *MACC1/β-catenin expression was not included in multivariable Cox analysis in this Table.

In our series, 211 samples (65.3%) were β-catenin abnormal expression, 112 samples (34.7%) were β-catenin normal expression. However, only 76 samples (23.5%) were β-catenin abnormal expression in ANM. β-catenin abnormal expression was significantly higher in CRC than that in ANM (p<0.001; Figure 2A, 2C). In the meantime, β-catenin abnormal expression rate in CRC with high MACC1 expression group was 82.2% (139/169), which was dramatically higher than 53.2% (82/154) of β-catenin normal expression rate in CRC with low MACC1 expression group. There was a significantly positive correlation between MACC1 expression and β-catenin abnormal expression in paraffin-embedded CRC tissues (r^2^=0.372, p<0.001; Table 2).

**Table 2:** The correlation between MACC1 expression and β-catenin expression in CRC tissue

Variable		MACC1 expression		P value	r2
		Low (%)	High (%)		
β-catenin expression	Normal	82 (25.4)	30(9.3)	<0.001	0.372
	Abnormal	72 (22.3)	139 (43.0)		

### Prognostic significance of MACC1 and β-catenin expression in CRC

Kaplan-Meier analysis showed that CRC patients had a significantly lower OS rate in the high MACC1 expression group compared with that in the low MACC1 expression group (p<0.001, Figure 2E). The OS rate for patients with high MACC1 expression at 3 years and 5 years was 67.8% (95% CI 60.7%~68.9%) and 41.5% (95% CI 33.7%~49.3%), respectively, compared with 96.7% (95% CI 93.9%~99.4%) and 82.8% (95% CI 77.0%~89.0%) for patients with low MACC1 expression at 3 years and 5 years, respectively. To determine whether MACC1 expression was an independent prognostic factor for CRC patients, univariate Cox regression analysis indicated that high MACC1 expression was significantly associated with reduced OS (HR=3.497; 95% CI 2.580~4.741; p<0.001) in CRC patients. In addition, other clinical parameters including histological differentiation, UICC stage, T, N, and M classification were also significant prognostic indicators for OS in CRC patients. Furthermore, multivariable Cox regression analysis demonstrated that high MACC1 expression was an independent prognostic indicator for reduced OS in CRC patients (p<0.001). In addition, histological differentiation, UICC stage, T, and N classification were independently associated with OS in CRC patients (Table 1).

We stratified our cohort of CRC patients into three groups according to the combination of different MACC1 and β-catenin expression levels: group 1, low MACC1/normal β-catenin expression (n=82); group 2, high MACC1/normal β-catenin expression and low MACC1/abnormal β-catenin expression (n=102); and group 3, high MACC1/abnormal β-catenin expression (n=139). For patients in group 1, the 3-year and 5-year OS was 100% and 93.8% (95% CI 88.5%~99.1%), respectively, which was significantly higher than 95.1% (95% CI 91.0%~99.2%) and 73.6(95% CI 65.0%~82.2%) for patients in group 2 (p<0.001) and 60.6% (95% CI 52.4%~68.8%) and 32.0% (95% CI 23.6%~40.4%) for patients in group 3, respectively (p<0.001; Figure 2F).

We further performed univariate Cox regression analysis. The patients in group 1 (low MACC1/normal β-catenin expression) was considered as the dummy variable (value=1). The patients in group 3 (HR=7.505; 95% CI 4.921~11.448; p<0.001) had a significantly higher hazard of death than the other groups. When the combination of MACC1 and β-catenin expression was considered as a single variable, univariate Cox regression analysis showed that high MACC1/abnormal β-catenin expression (HR=7.505 95% CI 4.921~11.448; p<0.001), MACC1 expression (HR=3.497 95% CI 2.580~4.741; p<0.001), histological differentiation (HR=3.650; 95% CI 2.385~5.586; p<0.001), UICC stage (HR=1.738 95% CI 1.295~2.333; p<0.001), T classification (HR=1.772 95% CI 1.257~2.499; p=0.001), N classification (HR=1.429 95% CI 1.073~1.904; p=0.015), and M classification(HR=1.522 95% CI 1.137~2.038; p=0.005) were independent prognostic factors for OS in CRC patients. Further multivariable Cox regression analysis demonstrated that high MACC1/abnormal β-catenin expression was the strongest independent prognostic indicator for reduced OS in CRC patients (HR=6.346; 95% CI 4.042~9.964; p<0.001). Histological differentiation (HR=1.775; 95% CI 1.125~2.801; p=0.014) and T classification (HR=1.558; 95% CI 1.088~2.232; p=0.016) were the other two important independent prognostic factors for OS in CRC patients. However, high MACC1 expression (HR=1.410; 95% CI 0.737~2.699; p=0.343), UICC stage (HR=1.142; 95% CI 0.816~1.599; p=0.331), N classification (HR=1.172; 95% CI 0.862~1.594; p=0.435), and M classification (HR=1.346; 95% CI 0.954~1.898; p=0.115) lost their statistical significance (Table 3).

**Table 3:** Multivariable Cox regression analysis for the prognostic value of clinicopathological parameters, MACC1 and β--catenin expression in CRC

Characteristics		Hazard ratio	95% CI	P value
Histological differentiation	Well	1.775	1.125~2.801	0.014
	Moderately			
	Poorly			
UICC stage	I- II	1.142	0.816~1.599	0.331
	III -IV			
T classification	T1 -T2	1.558	1.088~2.232	0.016
	T3 -T4			
N classification	N0	1.172	0.862~1.594	0.435
	N1 -N2			
M classification	M0	1.346	0.954~1.898	0.115
	M1			
MACC1 expression	Low	1.410	0.737~2.699	0.343
	High			
MACC1/β-catenin expression	Group1	6.346	4.042~9.964	<0.001
	Group2			
	Group3			

Group 1, Low MACC1/normal β-catenin expression; Group 2, Low MACC1/abnormal β-catenin + high MACC1/normal β-catenin expression; Group 3, High MACC1/abnormal β-catenin expression.

### MACC1 knockdown inhibited cell proliferation and colony formation in CRC cells

**Figure 3:**
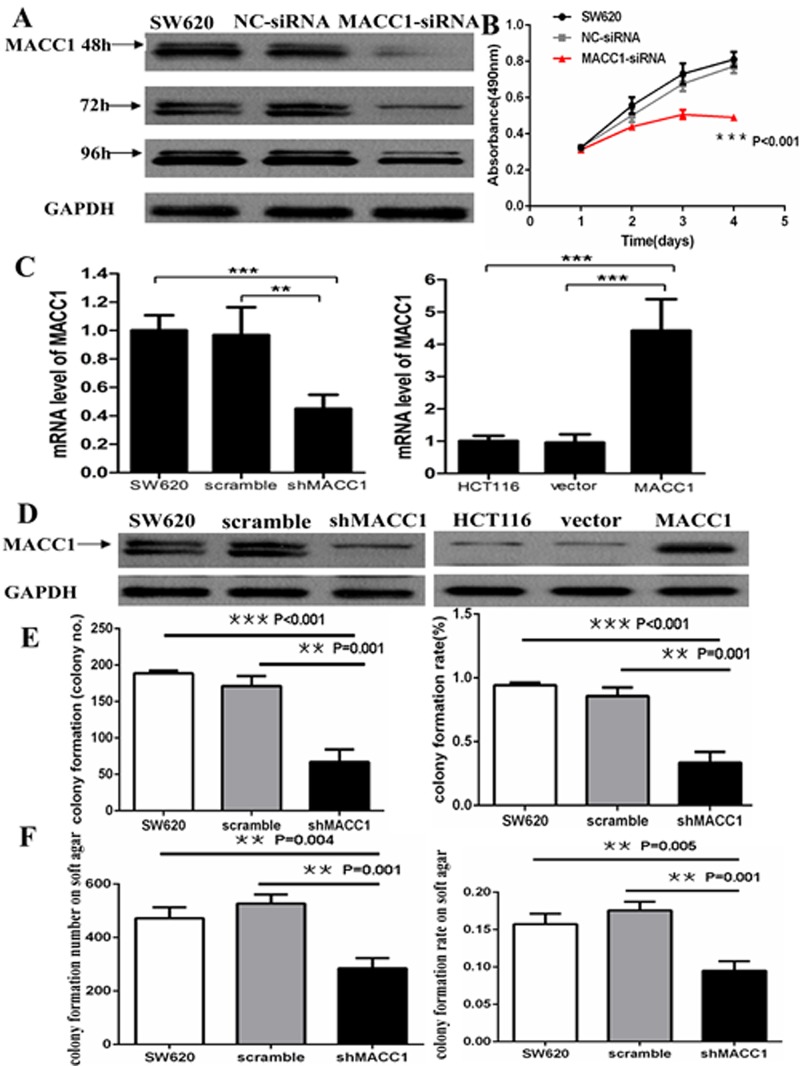
MACC1 protein expression reduced in SW620 cells transfected with MACC1-siRNA at 48, 72, and 96 hours. GAPDH was used as loading control (A); MACC1 knockdown dramatically suppressed cell proliferation of SW620 at 48, 72, and 96 hours compared with the scramble siRNA(NC-siRNA) and untreated SW620 group by MTT analysis, respectively (B); MACC1 mRNA and protein expression was suppressed in SW620 cells (left) stably transfected shMACC1 compared with the scramble and untreated control groups by real-time PCR and western blot analysis, respectively. MACC1 mRNA and protein expression was elevated in HCT116 cells (right) stably transfected with MACC1 expression plasmid compared with empty vector and untreated control groups, respectively. **P<0.01, ***P<0.001(C-D); MACC1 knockdown significantly inhibited colony formation (E) and soft agar colony formation (F) of SW620 cells, respectively. The histogram showed the relative mean number of colony number (left) and the colony formation rate (right) in SW620 cells.

MACC1 protein level in SW620 cells transfected with MACC1 siRNA was remarkablely decreased compared with the control group and the effect lasted up to 96 hours after transfection (Figure 3A). MTT assay showed that MACC1 knockdown significantly suppressed SW620 cell proliferation compared with the untreated and NC-siRNA transfection groups (p<0.001, Figure 3B). As shown in Figure 3C-D, MACC1 mRNA and protein level in SW620 cells stably transfected with shMACC1 was dramatically decreased more than 60% of the untreated and scrambled shRNA-transfection control groups using Real-time PCR and western blot analysis, respectively. Cell colony formation assay demonstrated that the mean number of colony formation in SW620 transfected with shMACC1 (mean number=67) was significantly less than that in untreated (mean number=189) and scrambled shRNA transfection (mean number=171) control groups (p<0.001 and p=0.001). The cell colony formation rate was also significantly suppressed about 60% in SW620 transfected with shMACC1 compared with the untreated and scrambled shRNA transfection control groups. Likewise, the colony formation on soft agar assay showed that much fewer colonies were found in SW620 cells transfected with shMACC1 (mean colony number=285) compared with untreated (mean colony number=475) and scrambled shRNA transfection (mean colony number=527) groups (p=0.004 and p=0.001). The colony formation rate on soft agar was also significantly suppressed in SW620 transfected with shMACC1 compared with the untreated and scrambled shRNA transfection control groups (p=0.005 and p=0.001), respectively (Figure 3E-F).

### MACC1 expression suppressed cell apoptosis in CRC cells

**Figure 4:**
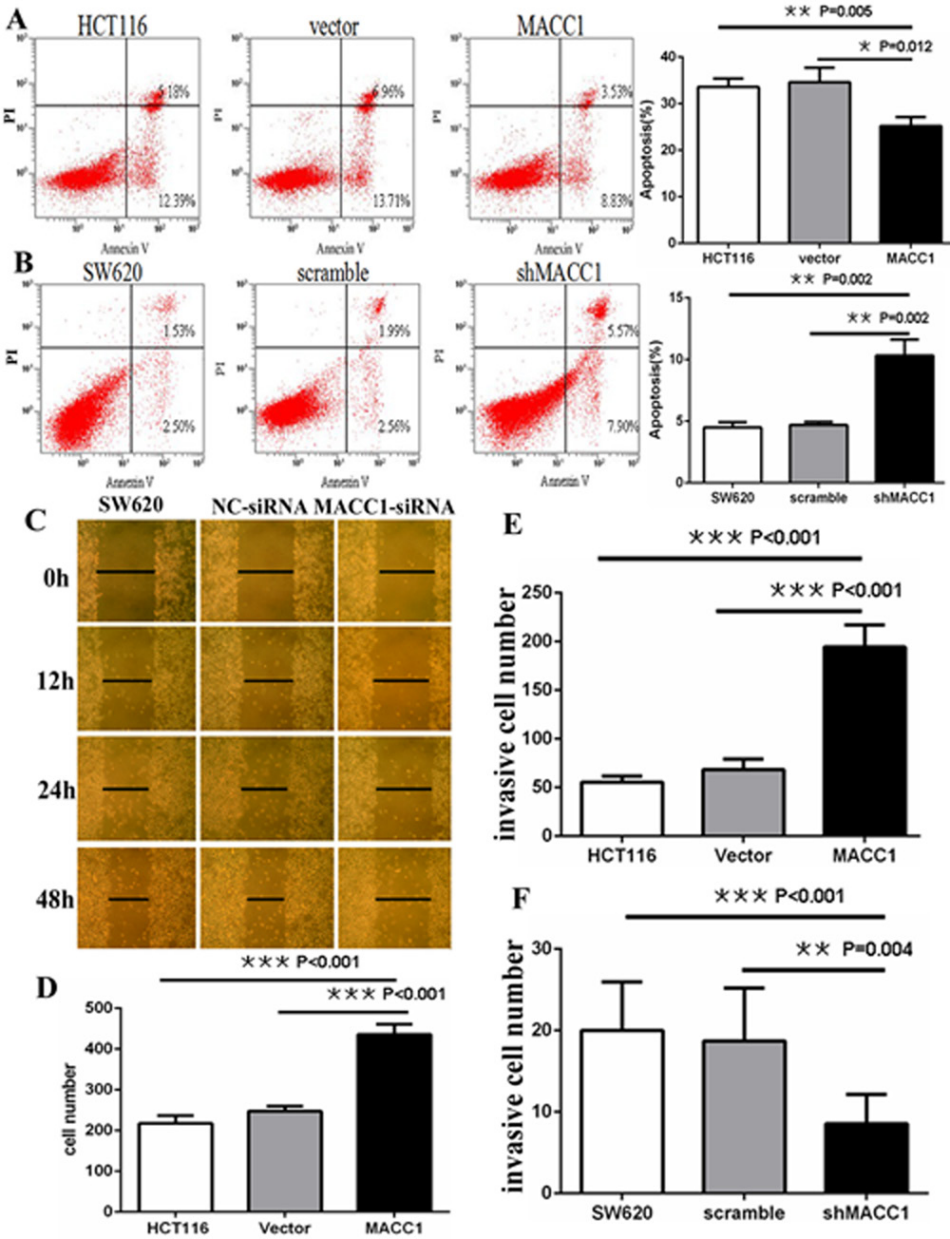
MACC1 inhibited or induced apoptosis in HCT116 cells (A) stably with MACC1 over-expression or SW620 cells (B) stably with shMACC1 compared with the control groups by flow cytometry analysis; MACC1 knockdown inhibited migration (C) and invasion (F) of SW620 cells compared with control groups by scratch wound assay and transwell invasion assay, respectively. MACC1 overexpression induced migration (D) and invasion (E) of HCT116 cells compared with control groups by transwell migration assay and transwell invasion assay, respectively.

As shown in Figure 3C-D, MACC1 mRNA and protein level in HCT116 stably transfected with MACC1 expression plasmid was significantly increased about 4.5-fold of the untreated and empty vector control groups using real-time PCR and western blot analysis, respectively. Flow cytometry analysis showed that cell apoptosis was significantly suppressed in HCT116 transfected with MACC1 over-expression plasmid compared with the untreated and vector-transfected control groups (p=0.005 and p=0.012), respectively. Likewise, cell apoptosis significantly increased in shMACC1-transfected SW620 cells compared with the untreated and scrambled shRNA-transfected control groups (p=0.002 and p=0.002), respectively (Figure 4A-B).

### MACC1 expression promoted migration and invasion of CRC cells

Two different experiments were carried out to clarify the effect of MACC1 expression on migration of CRC cells. As shown in Figure 4C, scratch wound assay showed that cell migration was dramatically inhibited in SW620 cells transfected with MACC1 siRNA compared with untreated and NC-siRNA-transfected control groups at 12, 24, and 48 hours, respectively. Furthermore, transwell migration assay showed that the mean number of migrated cells per field of view (mean number=436) was significantly more in HCT116 stably transfected with MACC1 expression plasmid than that in untreated (mean number=218) and empty vector (mean number=247) control groups (p<0.001 and p<0.001), respectively (Figure 4D). Transwell matrix penetration assay showed that the mean number of invasive cells (mean number=194) was significantly more in HCT116 stably transfected with MACC1 expression group than that in untreated (mean number=56) and empty vector (mean number=69) control groups (p<0.001 and p<0.001), respectively. Likewise, the mean number of invasive cells (mean number=9) was significantly fewer in SW620 cells stably transfected with shMACC1 group than that in untreated (mean number=20) and scrambled shRNA (mean number=19) control groups (p<0.001 and p=0.004), respectively (Figure 4E-F).

### MACC1 promoted the tumorigenicity of CRC cells *in vivo*

**Figure 5:**
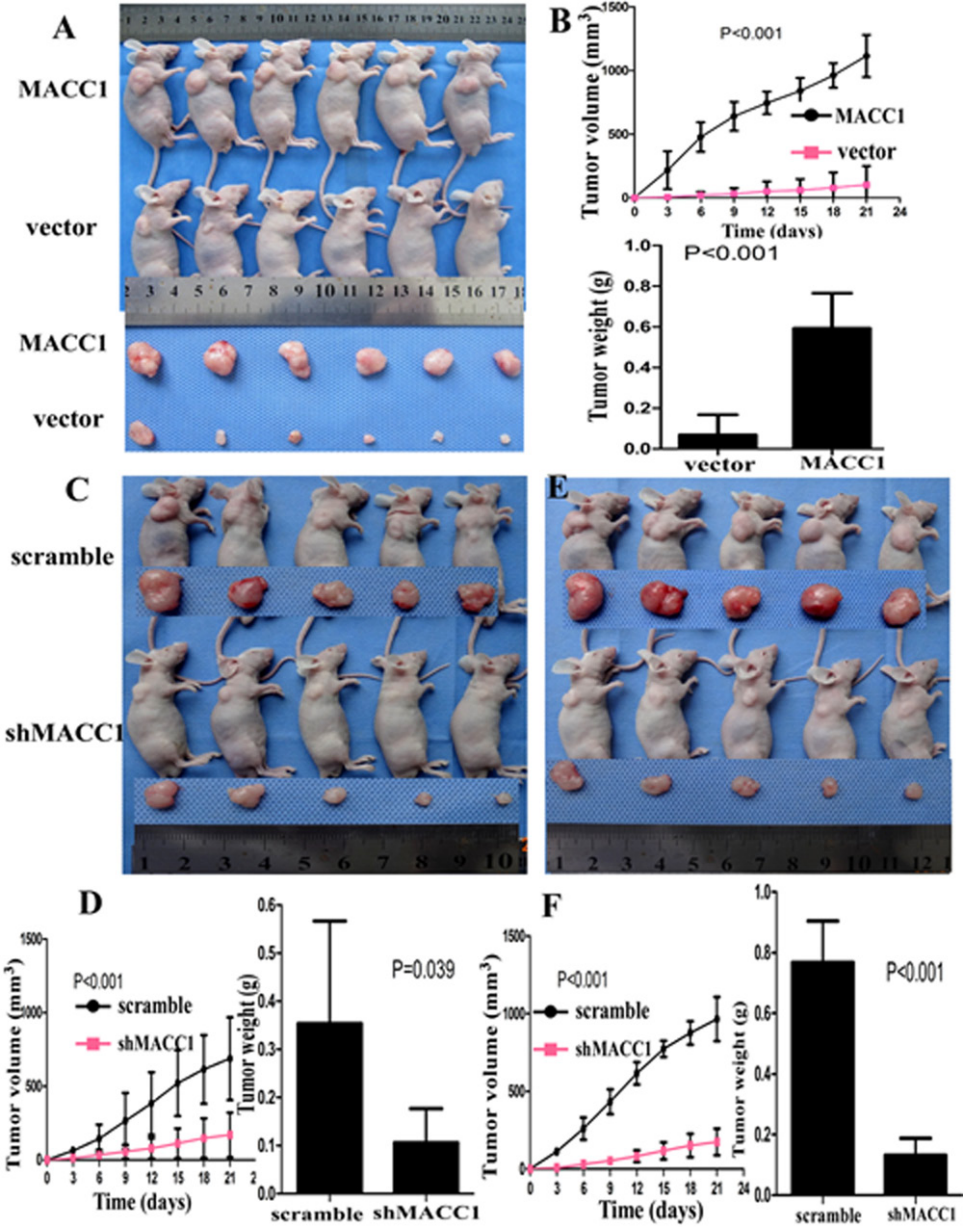
MACC1 over-expression significantly promoted tumor growth of HCT116 cells implanted subcutaneously in BALB/c-nu mice compared with the control group (p<0.001; A, B-upper) and significantly increased tumor weight (P<0.001; B-lower). MACC1 knockdown significantly suppressed tumor growth of SW620 cells at a density of 1×10^6^/100μl (C-D) and 2×10^6^/100μl (E-F) implanted subcutaneously in BALB/c-nu mice compared with the control group and significantly reduced tumor weight, respectively.

To determine whether MACC1 affects the tumorigenicity of CRC cells in vivo, we performed tumor growth experiments in nude mice. Tumor formation was found at the third day and the sixth day in HCT116 cells stably transfected with MACC1over-expression plasmid group and empty vector control group after subcutaneous injection, respectively. As shown in Figure 5A-B, the average volume of tumors derived from HCT116 cells with MACC1 over-expression group was dramatically larger than that of empty vector control group (p<0.001). Tumor with MACC1 over-expression group exhibited a rapid increase (approximately 12-fold) in tumor volume over 21 days. However, tumor with empty vector control group showed only a 2-fold increase in tumor volume over 21 days. Concomitantly, the average final tumor weight in MACC1-transfected group (0.59 g) was significantly more than that in empty vector control group (0.08g) upon termination of the experiment (p<0.001).

This finding was further confirmed in SW620. When SW620 cells were injected at density of 1×10^6^/100μl, tumor formation was found at the sixth day and the third day in SW620 stably transfected with shMACC1 group and scrambled shRNA control group after subcutaneous injection, respectively. The tumor volume of shMACC1-transfected group increased approximately 1.8-fold over 21 days while scrambled shRNA control group showed a 7-fold increase (p<0.001). The average final tumor weight in shMACC1-transfected group (0.11g) was much less than that in scrambled shRNA control group (0.36g) upon termination of the experiment (p<0.001; Figure 5C-D). When SW620 cells were injected at density of 2×10^6^/100μl, tumor formation was found at the fifth day and the third day in shMACC1-transfected group and scrambled shRNA control group after subcutaneous injection, respectively. The tumor volume of shMACC1-transfected group increased approximately 2.3-fold over 21 days while scrambled shRNA control group showed a 9.5-fold increase (p<0.001). The average final tumor weight in shMACC1-transfected group (0.13 g) was also dramatically less than that in scrambled shRNA control group (0.77g) upon termination of the experiment (p=0.039; Figure 5E-F).

**Figure 6:**
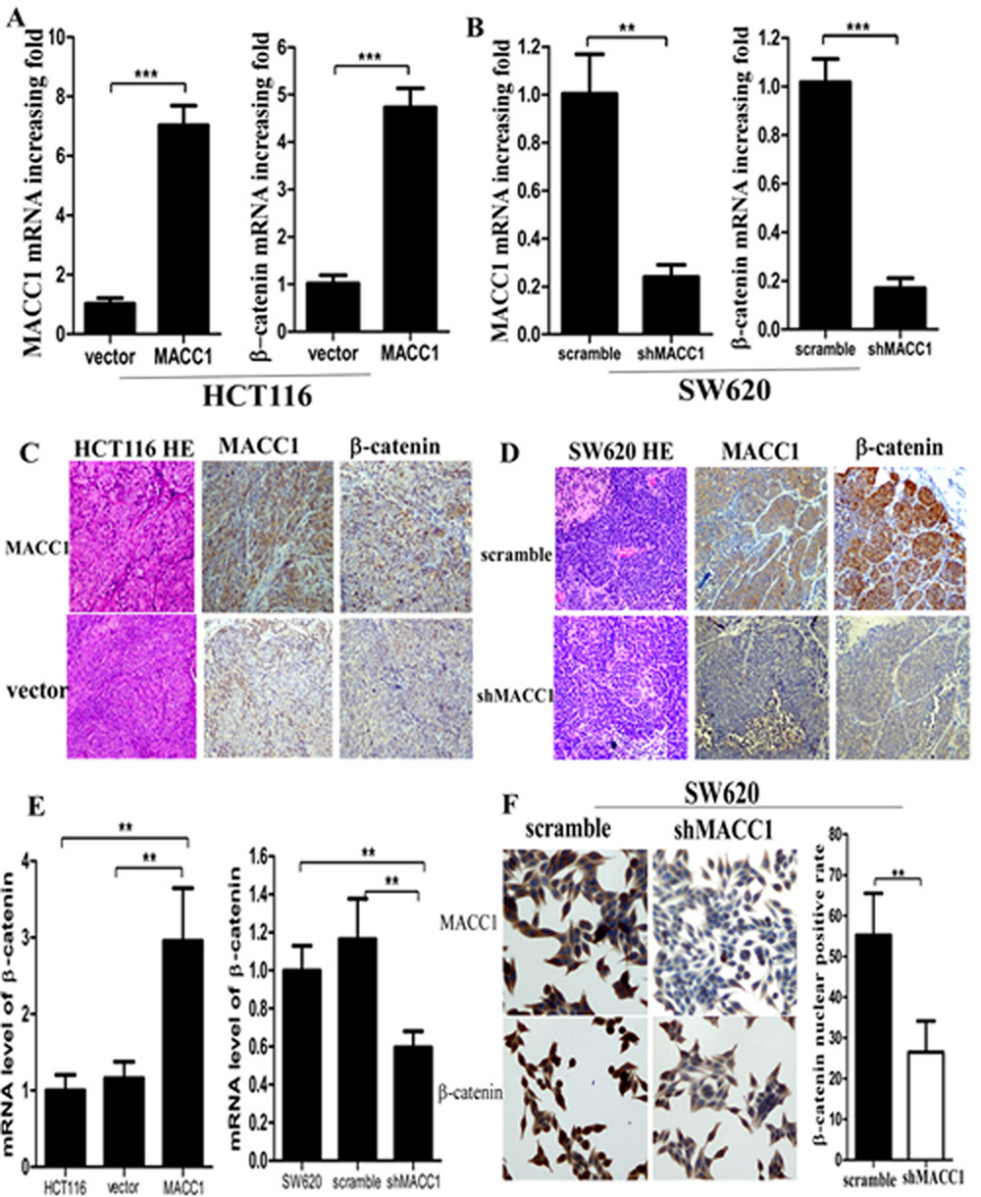
MACC1 and β-catenin mRNA (A-B) and protein (C-D) expression were much increased or decreased in enucleated tumors derived from HCT116 cells stably transfected with MACC1 over-expression plasmid or SW620 stably transfected with shMACC1 compared with the control group by real-time PCR and immunohistochemistry staining (×400), respectively; β-catenin mRNA expression was dramatically increased or decreased in HCT116 with MACC1 over-expression or SW620 cells with shMACC1 compared with the control groups by real-time PCR analysis (E); MACC1 and nuclear β-catenin protein expression was dramatically decreased in SW620 cells with shMACC1 compared with the control group by immunohistochemistry staining, ×400. **p=0.004 (F).

Moreover, MACC1 and β-catenin mRNA and protein expression were much increased in enucleated tumors derived from HCT116 cells stably transfected with MACC1 over-expression plasmid compared with the empty vector control group by real-time PCR and immunohistochemistry staining, respectively. Likewise, MACC1 and β-catenin mRNA and protein expression was much decreased in enucleated tumors derived from SW620 cells stably transfected with shMACC1 compared with scrambled shRNA control group, respectively (Figure 6A-D).

### MACC1 expression increased β-catenin signaling in CRC cells

**Figure 7:**
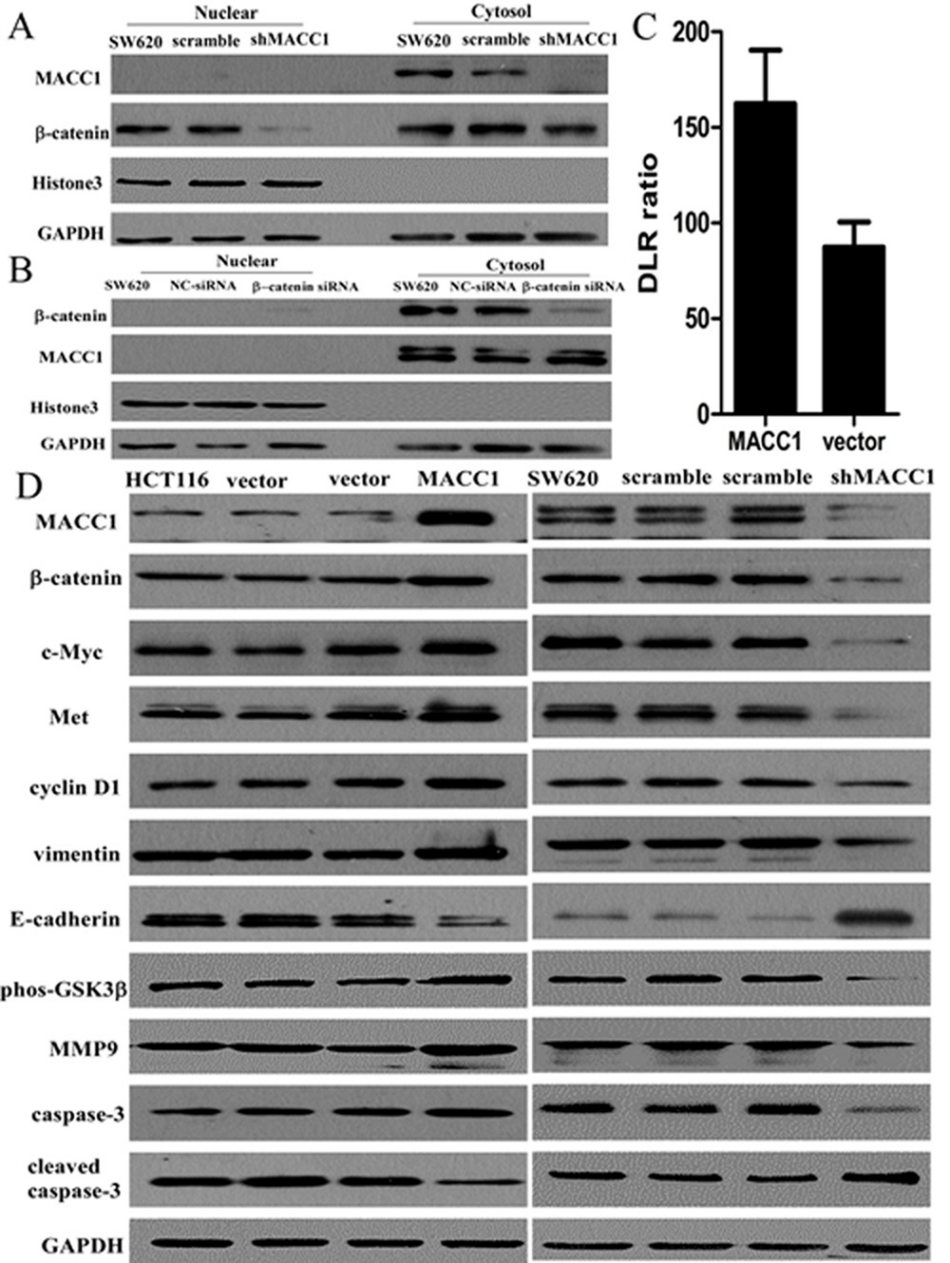
Nuclear β-catenin expression was suppressed in SW620 cells with shMACC1 compared with the control groups by western blot analysis (A); β-catenin knockdown had no remarkable effect on MACC1 protein expression in SW620 cells transfected with β-catenin-siRNA compared with the control group by western blot analysis (B). GAPDH level was considered loading control, Histone3 was considered as nuclear loading control. MACC1 expression significantly increased β-catenin transcriptional activity in SW620 cells transfected with MACC1 expression plasmid at 48 hours compared with the empty vector control group by dual-luciferase reporter assay (p=0.014; C). MACC1 expression increased Met, c-Myc, cyclin D1, MMP9, phos-GSK-3β (Ser9), and vimentin expression, but suppressed cleaved caspase-3 and E-cadherin expression in HCT116 cells transfected with MACC1 over-expression plasmid compared with the control group by western blot analysis. However, MACC1 knockdown reversed all these changes in SW620 cells by western blot analysis (D).

As shown in Figure 6E and Figure 7, MACC1 over-expression dramatically increased β-catenin mRNA and protein expression in HCT116 cells compared with the empty vector control group by real-time PCR and western blot analysis, respectively. Likewise, MACC1 knockdown significantly decreased β-catenin mRNA and protein expression in SW620 cells compared with the control group, respectively.

Further study showed that MACC1 knockdown significantly reduced nuclear β-catenin accumulation in SW620 cells compared with the control group by immunocytochemistry staining (p=0.004, Figure 6F). To further determine if there was increased nuclear β-catenin expression in CRC cells with MACC1 expression, SW620 cells transfected with shMACC1 were fractionated into cytoplasmic and nuclear fractions and β-catenin levels were determined by western blot analysis. Decreased nuclear β-catenin expression level was found in SW620 with shMACC1 compared with the control group (Figure 7A). However, β-catenin knockdown had no remarkable effect on MACC1 expression in SW620 cells transfected with β-catenin siRNA at 48 hours compared with the control group by cellular fractionation and western blot analysis (Figure 7B). To further determine whether nuclear β-catenin accumulation in SW620 cells induced by MACC1 expression was transcriptionally activated, dual-luciferase reporter assay showed that transcriptional activity of β-catenin significantly increased in SW620 cells transfected with MACC1 expression plasmid compared with the empty vector control group at 48 hours (p=0.014, Figure 7C).

Further mechanism study showed that MACC1 expression increased Met, β-catenin down-stream genes including c-Myc, cyclin D1, and MMP9, and up-stream gene phos-GSK-3β (Ser9) expression, suppressed cleaved caspase-3 expression in HCT116 cells transfected with MACC1 over-expression plasmid compared with the control group by western blot analysis. Likewise, MACC1 knockdown suppressed Met, β-catenin down-stream genes including c-Myc, cyclin D1, and MMP9, and up-stream gene phos-GSK-3β (Ser9) expression, increased cleaved caspase-3 expression in SW620 cells with shMACC1 compared with the control group by western blot analysis. Wang et al. have reported MACC1 overexpression upregulated mesenchymal-epithelial transition (EMT) factor and induced changes to markers of EMT in gastric cancer. Our data showed that vimentin expression was increased and E-cadherin expression was suppressed in HCT116 with MACC1 over-expression compared with the control group by western blot analysis. However, vimentin expression was suppressed and E-cadherin expression was increased in SW620 cells with shMACC1 compared with the control group by western blot analysis (Figure 7D).

## DISCUSSION

Our results showed that MACC1 expression was significantly related to histological differentiation, UICC stage, T classification, and N classification of CRC. High MACC1 expression was an independent prognostic indicator for reduced OS in CRC patients. Previous reports have showed that overexpression of MACC1 potentiates metastasis and recurrence of CRC[15], and associates with peritoneal dissemination and higher stage of TNM classification in CRC [16]. Shirahata et al. have reported that MACC1 expression shows significant correlation with peritoneal dissemination of gastric carcinoma [17, 18]. However, Ge et al. study shows that over-expression of MACC1 associates with better prognosis of gastric cancer patients [19]. The difference may be related to the different evaluation criteria for MACC1 expression.

Gao et al. reported that knockdown of MACC1 expression suppressed hepatocellular carcinoma cell migration and invasion and inhibited expression of MMP2 and MMP9 [5]. Down-regulation of MACC1 resulted in significant inhibition of cell proliferation, migration and invasion, meanwhile obvious enhancement of apoptosis in ovary carcinoma cells [6]. Our recent study shows that MACC1 down-regulation inhibits proliferation and tumorigenicity of nasopharyngeal carcinoma cells [9]. In order to investigate the role of MACC1 in carcinogenesis of CRC, our data showed that MACC1 knockdown dramatically inhibited cellular proliferation, migration, invasion, colony formation, and tumorigenesis, both in vitro and in vivo, but induced apoptosis in CRC cells. Further study showed that MACC1 knockdown increased promoting apoptosis protein-cleaved caspase-3 and suppressed cellular migration and invasion factor-MMP9 expression in SW620 cells with shMACC1.

To determine the relationship between MACC1 and β-catenin expression in CRC, our study showed that significant positive correlation between MACC1 expression and abnormal β-catenin expression was found in CRC tissues (p<0.001). High MACC1/abnormal β-catenin expression was the strongest independent prognostic factor for reduced OS in CRC patients (p<0.001). Further mechanism study showed that MACC1 over-expression increased β-catenin and its downstream genes including c-Myc, cyclin D1, and MMP9 expression in CRC cells. MACC1 is a newly identified key regulator of HGF-MET signaling in colorectal carcinoma [2, 3]. Consistent with such report, our data demonstrated that MACC1 over-expression or knockdown increased or suppressed Met expression in HCT116 or SW620 cells. Boon et al. have reported that Met expression is part of a genetic program controlled by the Wnt/β-catenin pathway in differentiation of intestinal epithelium and CRC [20]. GSK3β could be inactivated by phosphorylation at Ser-9 by serine/threonine kinases, such as Akt, protein kinase A, and protein kinase C, followed by decreased activities [21, 22]. Phosphorylation of β-catenin by GSK-3β results in ubiquitin-mediated degradation of β-catenin [11]. Our data showed that phos-GSK3β(Ser9) was indeed decreased in SW620 transfected with shMACC1, which meant that GSK3β activity was increased by MACC1 knockdown. The expression pattern of phos-GSK3β was similar to that of β-catenin, leading us to postulate that the β-catenin degradation is attributed to the increased GSK3β activity induced by MACC1 knockdown. In fact, decreased GSK3β activity and corresponding upregulation of β-catenin were also seen in HCT116 cells with MACC1 over-expression.

Wang et al. have reported MACC1 overexpression upregulated mesenchymal-epithelial transition (EMT) factor and induced changes to markers of EMT in gastric cancer [7]. Our data showed that MACC1 over-expression increased EMT marker-vimentin expression and suppressed E-cadherin expression in HCT116 cells. Whereas silencing of MACC1 reversed all these changes.

In summary, our study suggest that MACC1/GSK3β/β-catenin signaling pathway might play an important role in carcinogenesis of CRC and contributes to progression of CRC through EMT.

## MATERIALS AND METHODS

### Patient Information

Twelve pairs of fresh CRC tissues and adjacent non-tumour colorectal mucosa (ANM) tissues, 323 pairs of paraffin-embedded, archived CRC and ANM tissues between 2002 and 2006 were collected from Department of Pathology, the first Affiliated Hospital, Sun Yat-sen University, Guangzhou, China. No patient had received chemotherapy or radiotherapy before surgery. The histopathology of the disease was determined by two pathologists according to the criteria of the World Health Organization. Clinical staging was done according to the Union for International Cancer Control (UICC) classification. For the research purposes of these clinical materials, prior patient's consents and approval from the Institutional Research Ethics Committee were obtained. Follow-up information was available for all patients. Detailed clinical information is summarized in Table 1.

### Cell lines and small interfering RNA (siRNA) sequences

The human CRC cell lines SW480 and SW620 were maintained in Leibovitz's L-15 Medium (Invitrogen, Carlsbad, CA), HCT116 was grown in McCoy's 5A Medium (Invitrogen). LOVO and SW1116 were cultured in RPMI-1640 medium (Invitrogen). HT29 was maintained in Dulbecco's modified Eagle's medium (Invitrogen). The human colonic epithelial cell line NCM460 was cultured in RPMI-1640 medium. All medium were supplemented with 10% (v/v) fetal bovine serum (Invitrogen), 1×antibiotic/antimycotic (100 units/mL streptomycin, 100 units/mL penicillin, and 0.25 mg/mL amphotericin B). All cell lines were cultured in humidified incubator at 37°C with 5% CO_2_.

The targeted MACC1 sequences were: sense 5'-CAC CAU AGC UUG CAA AGU A dTdT-3', antisense 5'-UAC UUU GCA AGC UAU GGU G dTdT-3'. The targeted β-catenin sequences were: sense 5'-GCC ACA AGA UUA CAA GAA A dTdT-3', antisense 5'-UUU CUU GUA AUC UUG UGG C dTdT-3'. The siRNA duplexes were chemically synthesized and purified by Ribobio Inc. (Guangzhou, China). The siRNA was transfected using Lipofectamine RNAiMAX transfection reagent (Invitrogen) according to the manufacturer's instructions. Lipofectamine RNAiMAX alone and scrambled siRNA (NC-siRNA) were used as negative control groups.

### Establishment of stably transfected cell lines

For MACC1 over-expression, ectopic MACC1 coding sequence was amplified by polymerase chain reaction (PCR). The primer sequences were: forward: 5'-CCG CTC GAG ATG CTA ATC ACT GAA AGA AAA C-3'; reverse: 5'-CCG CTC GAG CTA TAC TTC CTC AGA AGT GGA GAA T-3'. The amplified product was cloned into the pBaBb-puromycin plasmid and confirmed by sequencing. For MACC1 silencing, sequences of short hairpin RNA targeting MACC1 (shMACC1) and scrambled siRNA were cloned into the pSUPER-retro-puromycin plasmid, respectively. The shMACC1 sequences were: 5'-TTC ACC CTT CGT GGT AAT AAT-3', and the scrambled siRNA sequences were: 5'-CAA CAA GAT GAA GAG CAC CAA-3'. CRC cell lines were transfected with aforementioned constructed plasmids or empty vector. Stably transfected cell lines were selected with 0.5 μg/ml puromycin at 48 hours after infection.

### Real-time PCR analysis

As described previously [9], the primer sequences used for MACC1 were purchased from TIANGEN BIOTECH (Beijing, China) CO., LTD. and followed: forward: 5' TTC TTT TGA TTC CTC CGG TGA; reverse: 5' ACT CTG ATG GGC ATG TGC TG. The primer sequences used for β-catenin were followed: forward: 5' TTG AAA ATC CAG CGT GGA CA; reverse: 5' TCG AGT CAT TGC ATA CTG TC. The geometric mean of housekeeping gene GAPDH was used to normalize the variability at mRNA expression levels. All experiments were performed in triplicate.

### Cell proliferation assay

8×10^3^ cells were seeded in each well of 96-well plate and incubated overnight. After transfection with 100 nM MACC1 siRNA, cell proliferation was determined at 0, 24, 48, and 72 hours using the 3-(4,5-dimethyl thiazol-2-yl)-2,5-diphenyl tetrazolium bromide (MTT) assay (CellTiter 96 Non-Radioactive Cell Proliferation Assay Kit (Promega Corporation, Madison, WI). Cells transfected with NC-siRNA and untreated cells were used as the control groups. This experiment was performed in triplicate.

### Colony formation assay

200 SW620 cells with stably transfected shMACC1 were plated onto 6-well plates and cultured for 2 weeks. Fresh medium was added every 4 days. At the end-point, the cells were washed lightly with cold phosphate buffered saline (PBS) twice, fixed with 4% paraformaldehyde for 30 minutes and stained with 1% crystal violet solution for 20 minutes at room temperature. The number of colonies in 10 random view fields was counted under a microscope and the average number of colonies was achieved. Scrambled NC-siRNA and untreated cells were used as the control groups. The experiment was triplicated independently.

### Soft agar colony formation assay

3.0×10^3^ SW620 cells with shMACC1 in 3 ml 0.4% agarose with 2×L-15 medium were plated on top of 3 ml 0.6% bottom agarose existing in 6-well tissue culture plates. The plates were incubated at 37°C in a 5% CO_2_ incubator for 3 weeks and 200μl fresh medium was added to the top layer every 3 days. At the end-point, colonies were examined under a microscope at ×40 magnification and only colonies containing >50 cells were counted. Scrambled NC-siRNA and untreated cells were used as the control groups. Each treatment was performed in triplicate.

### Apoptosis assay

2×10^5^ cells were collected and washed twice with cold PBS, then resuspended in 500 μl cold Annexin binding buffer containing 5 μl Annexin V-FITC and 5 μl propidium iodide. The cells were incubated for 15 minutes in the dark at room temperature and analyzed using a Becton Dickinson FACScan (Becton Dickinson Immunocytometry Systems, San Jose, CA). Scrambled NC-siRNA, empty vector, and untreated cells were used as the control groups. The experiment was triplicated independently.

### Scratch wound assay

Cells were plated in 6-well plates and incubated overnight until 30%-50% confluent, then transfected with 100 nM MACC1 siRNA. Scrambled siRNA and untreated cells were used as the control groups. When cells were grown to confluency, vertical scratches were then made using a 200 μl plastic filter tip to create a ‘wound’ of approximately 200 μm in diameter. To eliminate dislodged cells, culture medium was removed and wells were washed with PBS. ‘Wound closure’ was observed at 0, 12, 24, 48 hours and digital images were taken under an inverted microscope.

### Transwell migration and invasion assays

15×10^4^ SW620 cells and 6×10^4^ HCT116 cells were plated in serum-free media in the upper chamber of 24-well Transwell Chambers (Corning Incorporated, Life Sciences, NY, USA), respectively, while media containing 10% FBS was added to the lower chamber as chemoattractant. Cells were allowed to invade through the matrigel (BD Biosciences) or migrate for 24 hours at 37°C with 5% CO_2_. Following invasion or migration, cells were fixed with 4% formaldehyde and stained with crystal violet. Cells on the upper surface of the filters were removed by wiping with a cotton swab. Migratory or invasive cells on the lower membrane surface were fixed in 1% paraformaldehyde, stained with hematoxylin, and counted (Ten random 200×fields per well). Cell counts were the mean number of cells per field of view. Three independent experiments were performed and the data were presented as mean ± standard deviation (SD).

### Western blot analysis

25μg of protein was loaded and separated in 10% sodium dodecyl sulfate polyacrylamide gel electrophoresis (SDS-PAGE) gel and transferred to polyvinylidine difluoride membranes (Millipore, Bedford, MA). The following antibodies were used to probe the alterations of protein: MACC1 (Sigma, St. Louis, MO),β-catenin, cyclin D1, vimentin, E-cadherin, phosphorylated-GSK3β (phos-GSK3β) (Ser9), MMP9 (Cell Signaling Technology, Danvers, MA),c-Myc, Met, total caspase-3, and cleaved caspase-3 (Santa Cruz Biotechnology, Santa Cruz, CA), Signal was detected by enhanced chemoluminescence techniques (Millipore). GAPDH or Histone 3 (Cell Signaling Technology) was used as the loading control.

### Luciferase reporter assay

For dual-luciferase reporter assay, SW620 cell lines were transiently cotransfected with 0.4μg of T cell factor (TCF) reporter plasmid TOPFlash which contains the luciferase gene under the control of a promoter with three wild-type TCF-responsive sites (Upstate Biotechnology, Lake Placid, NY) and 0.4μg of MACC1 over-expression effector plasmid. To control the transfection efficiency, a control reporter, pRL-TK (0.4μg), which contains a herpes simplex virus thymidine kinase promoter driving a Renilla luciferase gene, was cotransfected. After 48 hours, cells were lysed in passive lysis buffer, and luciferase activities were monitored in cell lysate with the use of dual-luciferase assay reagents (Promega) as described by the manufacturer, respectively. All reporter assay results presented were from two independent experiments prepared in triplicate.

### Immunohistochemistry staining

Three hundred twenty three pairs of paraffin-embedded CRC and ANM tissues were made into the tissue microarrays.

As described previously [9], the primary antibodies including MACC1 (Sigma, St. Louis, MO, 1:200) and β-catenin (Santa Cruz Biotechnology, Santa Cruz, CA, 1:200) were used. The degree of MACC1 staining was based on both the proportion of positively stained tumor cells and intensity of staining. We evaluated MACC1 expression in CRC specimen by determining the staining index, which scores as 0, 1, 2, 3, 4, 6, 8, 9, and 12. The staining index score of 6 (the cutoff point) was used to distinguish between low and high expression of MACC1. The staining of β-catenin was scored according to Maruyama's method[23]. When more than 70% of carcinoma cells were positively stained for membranous β-catenin, the cells was classified as β-catenin normal expression; if more than 10% of carcinoma cells were positively stained for cytoplasm or nuclei was regarded as β-catenin abnormal expression.

### Xenograft tumor model

Male BALB/c-nude mice (4-5 weeks old and weighing 15-16g) were housed under pathogen-free conditions. The mice were randomly assigned into treatment group and negative control group. Cells were trypsinized, washed twice with serum-free medium and reconstituted in serum-free medium mixed 1:1 with Matrigel (Becton-Dickinson) and then inoculated subcutaneously into the right flank of each nude mouse. MACC1-transfected HCT116 cell lines were injected at a density of 2×10^6^/100μl, empty vector-transfected HCT116 cell line was used as control group. SW620 cells with shMACC1 were injected at a density of 1×10^6^/100μl and 2×10^6^/100μl, respectively. Scrambled siRNA- transfected SW620 cell line was used as the control group. The treatment time was 21 days. The mice were monitored daily for adverse affects. Tumor size was measured every 3 days using a digital caliper, and the tumor volume was calculated according to the formula: tumor volume (mm^3^)=length×width^2^×0.5. At the end of the experiment, all mice were sacrificed and the total weight, tumor weight, and tumor volume were recorded. All the experiments were performed following the Guide for the Care and Use of Laboratory Animals (National Institutes of Health publication).

### Statistical Analyses

Chi-square test was used to compare the levels of MACC1 and β-catenin expression with different groups and various clinicopathological parameters. The Kaplan-Meier survival curves were used to estimate overall survival (OS). The significance of predictor variables for OS was evaluated by the log-rank test. Prognostic factors associated with OS were investigated according to the Cox proportional hazards regression model in a stepwise manner. Only those factors that were statistically significant (p<0.05) in the univariate survival analysis were included in the multivariate analyses. Groups from cell culture and in vivo experiments were compared using an unpaired, two-tailed Student's t tests and results were presented as means±SD. For MTT assay, comparison was done by univariate variance analysis (two-way ANOVA). Statistical analyses were performed using SPSS 16.0 statistical software. P<0.05 was considered to be statistically significant.
